# Atrial Septostomy and Left Ventricular Venting During Peripheral VA-ECMO

**DOI:** 10.1016/j.jacadv.2026.103344

**Published:** 2026-10-17

**Authors:** Arjun B. Kapoor, Joy Stouffer, Dhruv Sarma, Nanda Nayak, Parth Visrodia, Brandon Petree, Enock Adjei, Amir Teimouri Dereshgi, Emily N. Hollis, Jared O'Leary, Bret Alvis, Aaron Williams, Matthew Bacchetta, Aniket S. Rali

**Affiliations:** a Division of Cardiovascular Diseases, Vanderbilt University Medical Center, Nashville, Tennessee, USA; b Department of Medicine, Vanderbilt University Medical Center, Nashville, Tennessee, USA; c Department of Thoracic Surgery, Vanderbilt University Medical Center, Nashville, Tennessee, USA; d Department of Cardiac Surgery, Vanderbilt University Medical Center, Nashville, Tennessee, USA; e Department of Anesthesia, Vanderbilt University Medical Center, Nashville, Tennessee, USA; f Department of Biomedical Engineering, Vanderbilt University, Nashville, Tennessee, USA

**Keywords:** balloon atrial septostomy, cardiogenic shock, extracorporeal membrane oxygenation, left atrial decompression, left ventricular venting, mechanical circulatory support

## Abstract

**Background:**

Peripheral venoarterial (VA)–extracorporeal membrane oxygenation (ECMO) increases left ventricular (LV) afterload, and therefore the risk of LV distension, pulmonary edema, and cardiac stasis. Balloon atrial septostomy (BAS) provides atrial-level unloading, but the durability of LV venting remains uncertain.

**Objectives:**

This study aimed to evaluate the clinical effectiveness of BAS for LV venting in patients requiring peripheral VA-ECMO for cardiogenic shock (CS).

**Methods:**

The authors conducted a retrospective study of consecutive adults with CS supported with peripheral VA-ECMO who underwent BAS for LV unloading at an academic center (2019-2024). The primary endpoint was LV venting failure, a composite of pulmonary edema, LV distension, spontaneous echocardiographic contrast, LV or aortic root thrombus, stroke due to ECMO, or escalation to additional mechanical support.

**Results:**

A total of 28 patients were included in the study. BAS resulted in a significant reduction in mean left atrial pressure (20.5-13.5 mm Hg; *P* < 0.001). Durable LV venting was achieved in a subset of patients; however, LV venting failure occurred in 53.6%, driven primarily by pulmonary edema. Pulse-pressure trajectories differed according to eventual LV venting-failure status (interaction *P* = 0.018). Model-estimated pulse pressure was higher at 48 hours (*P* = 0.010) and 72 hours (*P* = 0.031) among patients who ultimately did not experience venting failure.

**Conclusions:**

Among patients receiving peripheral VA-ECMO for CS, BAS achieves atrial-level decompression and may provide durable LV venting in select patients, although venting failure remains common. Observed arterial pulse-pressure trajectories differed according to eventual LV venting failure status.

Peripheral venoarterial (VA)–extracorporeal membrane oxygenation (ECMO) provides comprehensive circulatory and pulmonary support for patients with refractory cardiogenic shock (CS) but increases left ventricular (LV) afterload through retrograde aortic flow.^1^ This heightened afterload predisposes the ailing heart to LV distension, pulmonary edema, intracardiac thrombus formation, and impaired myocardial recovery.^1^^,^^2^ Prior research has demonstrated that LV unloading strategies are associated with reduced mortality in VA-ECMO patients.^3^ Although LV unloading refers to reducing LV volume and pressure to lessen wall stress and myocardial oxygen demand, LV venting focuses on lowering LV and left atrial (LA) volumes to relieve pulmonary congestion and prevent blood stasis in the LV and aortic root.^2^^,^^4^ In practice, many unloading strategies simultaneously achieve venting, and the extent to which a given approach accomplishes both objectives is critical for promoting myocardial recovery while minimizing secondary complications of LV stasis.^3^^,^^4^

Balloon atrial septostomy (BAS) is a percutaneous strategy for LA unloading that creates a left-to-right atrial shunt, indirectly decompressing the LV by reducing mean left atrial pressure (mLAP).^5^^,^^6^ Observational studies suggest that atrial-level unloading may improve outcomes in VA-ECMO, and BAS may also serve as a feasible venting strategy in select cohorts to reduce LV preload, but available series lack granular invasive hemodynamic and echocardiographic data, leaving the degree and durability of LV venting, as well as its impact on outcomes, uncertain.^5^, ^6^, ^7^, ^8^ Therefore, we sought to characterize hemodynamic and echocardiographic trajectories following BAS and describe the frequency and clinical manifestations of LV venting failure.

## Methods

### Study design and population

We conducted a retrospective observational study of consecutive adult patients (≥18 years of age) with CS who underwent peripheral VA-ECMO and BAS for LV unloading between November 2019 and April 2024 at a quaternary-care academic medical center (Vanderbilt University Medical Center, Nashville, Tennessee, USA). All VA-ECMO cannulations were performed by cardiothoracic surgeons, who remained involved in daily multidisciplinary shock-team management and decisions regarding initial and subsequent LV unloading strategies. ECMO circuit flow was titrated by the treating team according to clinical evidence of adequate systemic perfusion, including hemodynamic parameters, end-organ function, and biochemical markers. When BAS was selected for LV unloading, the procedure was performed by an interventional cardiologist. Baseline clinical characteristics and physiologic data were obtained through systematic review of the electronic medical record. Patients were excluded if a mechanical LV unloading device (eg, Impella) was used as the initial unloading strategy. The study was approved by the Vanderbilt University Medical Center Institutional Review Board.

### Data collection and variables

Baseline variables included demographics, comorbid conditions, laboratory values, and pre-ECMO invasive hemodynamic parameters. Atrial septostomy procedural characteristics included the imaging modality used for procedural guidance, final balloon size, and method used to confirm procedural success. Pre-BAS inotropic support was defined as the continuous administration of at least 1 of the following agents before BAS: dobutamine, milrinone, epinephrine, or dopamine. Post-ECMO cannulation variables included serial laboratory measurements and invasive hemodynamics collected at predefined time points. ECMO circuit flows at 4 and 24 hours after initiation were indexed to body surface area (BSA) by dividing each circuit flow (L/min) by BSA (m^2^) and were expressed in L/min/m^2^. Echocardiographic assessments were reviewed at baseline and subsequent time points to evaluate prespecified criteria comprising the LV venting-failure endpoint.

### Clinical endpoints

The primary endpoint was LV venting failure, defined as a composite of evidence of aortic root thrombus, echocardiographic evidence of LV distension, spontaneous echocardiographic contrast (SEC), evidence of LV thrombus, pulmonary edema, cerebrovascular accident due to ECMO, escalation to an intra-aortic balloon pump, or escalation to an Impella. Secondary endpoints included the individual components of the composite outcome, 30-day all-cause mortality, and survival to hospital discharge. LV distension was defined as either a documented increase in LV end-diastolic diameter on serial imaging or the presence of qualitative echocardiographic features of LV dilation and elevated filling pressures, as determined by 2 study team members via direct image review or formal report interpretation (including transthoracic or transesophageal echocardiography). SEC was either reported by the echocardiographer and confirmed by 1 study team member or confirmed by 2 study team members if not present on the formal report.

### Statistical analysis

Continuous variables are reported as median (Q1-Q3), unless otherwise specified, and categorical variables as counts and percentages. Longitudinal physiologic data were analyzed using linear mixed-effects models with random intercepts to account for within-patient correlation across predefined time points. Arterial pulse pressure was analyzed as a longitudinal physiologic variable using the same mixed-effects modeling framework, with baseline defined as the lowest pulse pressure in the 24 hours preceding VA-ECMO initiation. LV venting-failure status was used solely as an outcome-defined grouping variable for exploratory descriptive comparisons of observed longitudinal trajectories and was not treated as a baseline predictor. To assess potential bias from differential observation, the pulse-pressure mixed-effects model was repeated after excluding patients who died within 30 days without documented LV venting failure. Individual components of the composite endpoint were reported descriptively and were not modeled as predictors of the composite they defined. Pre- and post-BAS mLAP values were compared using a 2-sided Wilcoxon signed-rank test among patients with paired measurements. A 2-sided paired Student's *t*-test was performed as a sensitivity analysis. Analyses were performed using R, version 4.5.1 (R Core Team).

## Results

### Study population and baseline characteristics

Of the 284 adult patients with CS supported with VA-ECMO, 28 patients underwent BAS as their initial strategy for LV unloading and were included in the study (Supplemental Figure 1). Baseline demographic, clinical, laboratory, and hemodynamic characteristics are summarized in Table 1. The median age was 45.5 years (Q1-Q3: 39.0-57.3 years), and most patients were male (60.7%). Baseline cardiomyopathy was highly prevalent; 75.0% had a history of heart failure with reduced ejection fraction, and nonischemic cardiomyopathy was common (57.1%). Chronic kidney disease (53.6%) and chronic lung disease (25.0%) were frequent comorbidities, and atrial (50.0%) and ventricular arrhythmias (60.7%) were common.

**Table 1:** Baseline Demographic, Clinical, Laboratory, and Hemodynamic Characteristics

	Overall Cohort (N = 28)	LV Venting Failure (n = 15)	No LV Venting Failure (n = 13)
Age, y	45.5 (39-57.3)	44.0 (38.0-55.5)	46.0 (40.0-59.0)
Male	17 (60.7%)	10 (66.7%)	7 (53.8%)
Race			
White	16 (57.1%)	10 (66.7%)	6 (46.2%)
3Black	10 (35.7%)	4 (26.7%)	6 (46.2%)
Asian	1 (3.6%)	0 (0.0%)	1 (7.7%)
Other or unknown	1 (3.6%)	1 (6.7%)	0 (0.0%)
Hispanic ethnicity	0 (0.0%)	0 (0.0%)	0 (0.0%)
Weight, kg	78.4 (67.3-97.0)	74.8 (67.2-97.4)	81.2 (68.0-95.6)
Height, cm	173.9 (168.7-180.3)	175.3 (168.8-178.4)	172.7 (169.0-180.3)
BMI, kg/m^2^	27.8 (22.3-30.5)	29.7 (23.1-30.6)	27.2 (21.0-28.6)
BSA, m^2^	2.0 (1.8-2.2)	2.0 (1.8-2.2)	2.0 (1.9-2.2)
Baseline comorbidities			
Chronic liver disease	2 (7.1%)	0 (0.0%)	2 (15.4%)
Chronic kidney disease	15 (53.6%)	7 (46.7%)	8 (61.5%)
Congenital heart disease	2 (7.1%)	1 (6.7%)	1 (7.7%)
Known PFO/ASD	3 (10.7%)	1 (6.7%)	2 (15.4%)
Chronic lung disease	7 (25.0%)	2 (13.3%)	5 (38.5%)
Coronary artery disease	8 (28.6%)	2 (13.3%)	6 (46.2%)
HFrEF	21 (75.0%)	13 (86.7%)	8 (61.5%)
Ischemic cardiomyopathy	5 (17.9%)	2 (13.3%)	3 (23.1%)
Nonischemic cardiomyopathy	16 (57.1%)	12 (80.0%)	4 (30.8%)
HFpEF	4 (14.3%)	1 (6.7%)	3 (23.1%)
Hypertrophic cardiomyopathy	0 (0.0%)	0 (0.0%)	0 (0.0%)
Amyloid cardiomyopathy	0 (0.0%)	0 (0.0%)	0 (0.0%)
Sarcoid cardiomyopathy	0 (0.0%)	0 (0.0%)	0 (0.0%)
Severe aortic stenosis	1 (3.6%)	0 (0.0%)	1 (7.7%)
Severe aortic regurgitation	1 (3.6%)	1 (6.7%)	0 (0.0%)
Severe mitral regurgitation	5 (17.9%)	2 (13.3%)	3 (23.1%)
Severe tricuspid regurgitation	2 (7.1%)	1 (6.7%)	1 (7.7%)
Atrial arrhythmia	14 (50.0%)	9 (60.0%)	5 (38.5%)
Ventricular arrhythmia	17 (60.7%)	9 (60.0%)	8 (61.5%)
Severe carotid artery disease	1 (3.6%)	0 (0.0%)	1 (7.7%)
History of CVA	6 (21.4%)	2 (13.3%)	4 (30.8%)
Peripheral arterial disease	1 (3.6%)	0 (0.0%)	1 (7.7%)
Baseline laboratory values			
Sodium, mmol/L	133.5 (131-137)	133.0 (131.0-137.0)	134.0 (131.5-136.5)
Potassium, mmol/L	4.45 (3.9-4.7)	4.3 (3.9-4.7)	4.7 (4.3-5.2)
Chloride, mmol/L	99.0 (94.5-100.8)	99.0 (96.0-102.0)	100.0 (97.0-102.0)
Bicarbonate, mmol/L	21.0 (16.0-22.5)	21.0 (16.0-24.0)	21.0 (17.0-22.0)
BUN, mg/dL	21.0 (12.5-30.5)	20.0 (17.0-26.0)	23.0 (12.5-35.5)
Creatinine, mg/dL	1.29 (1.10-1.61)	1.2 (1.1-1.6)	1.4 (1.2-1.6)
Glucose, mg/dL	140 (92-244)	140.0 (116.0-194.0)	224.0 (96.5-282.5)
Calcium, mg/dL	8.6 (8.0-9.2)	8.7 (8.2-9.3)	8.5 (8.2-9.1)
Magnesium, mg/dL	2.6 (2.1-3.3)	2.5 (2.1-3.2)	2.6 (2.3-3.4)
Albumin, g/dL	3.35 (3.0-3.65)	3.3 (3.0-3.6)	3.3 (3.1-3.5)
Bilirubin, mg/dL	0.95 (0.55-1.3)	1.1 (0.7-1.3)	0.9 (0.6-1.5)
ALP, U/L	73.5 (60-111)	90.5 (61.5-123.8)	73.0 (71.0-86.2)
AST, U/L	54.5 (23-324)	54.5 (27.2-325.5)	51.0 (23.5-195.2)
ALT, U/L	42.0 (19-95)	42.0 (22.5-70.5)	52.0 (20.0-236.5)
BNP, pg/mL	1856 (432-4,788)	1855.5 (1,102.5-4,979.8)	2,624.0 (1913.5-3,334.5)
WBC, ×10^3^/μL	13.8 (9.5-17.9)	14.4 (9.3-16.2)	11.5 (8.6-20.1)
Hemoglobin, g/dL	11.1 (10.1-12.1)	11.1 (10.3-12.2)	11.0 (9.5-11.9)
Platelets, ×10^3^/μL	182 (119-244.0)	198.0 (93.0-249.0)	176.5 (139.8-228.2)
INR	1.55 (1.3-1.9)	1.5 (1.2-1.7)	1.8 (1.5-2.5)
Lactate, mmol/L	2.1 (0.9-5.2)	2.6 (1.4-5.2)	2.1 (0.9-5.9)
Hemodynamics			
SVR, dyn·s·cm^-5^	1,303.0 (966.3-1,494.0)	1,358.5 (1,129.0-1,464.0)	1,107.0 (900.2-1,365.8)
PA diastolic, mm Hg	32.0 (22.5-37.5)	33.0 (26.0-36.0)	23.0 (18.5-35.5)
PA systolic, mm Hg	47.0 (35.0-55.0)	46.5 (34.2-53.5)	47.0 (39.0-59.5)
Cardiac index, L/min/m^2^	2.0 (1.82-2.45)	1.9 (1.9-2.0)	2.3 (2.0-2.5)

Values are median (Q1-Q3) or n (%). Baseline characteristics of patients supported with peripheral VA-ECMO who underwent balloon atrial septostomy are presented overall and according to eventual LV venting failure status. Laboratory and hemodynamic values reflect measurements obtained before VA-ECMO initiation.

ALP = alkaline phosphatase; ALT = alanine transaminase; ASD = atrial septal defect; AST = aspartate aminotransferase; BMI = body mass index; BNP = B-type natriuretic peptide; BSA = body surface area; BUN = blood urea nitrogen; CVA = cerebrovascular accident; ECMO = extracorporeal membrane oxygenation; HFpEF = heart failure with preserved ejection fraction; HFrEF = heart failure with reduced ejection fraction; INR = International Normalized Ratio; LV = left ventricular; PA = pulmonary artery; PFO = patent foramen ovale; SVR = systemic vascular resistance; VA = venoarterial; WBC = white blood cell.

### VA-ECMO support and balloon atrial septostomy procedural characteristics

VA-ECMO support and procedural characteristics are summarized in Table 2. The median ECMO run time was 79.0 hours (Q1-Q3: 54.5-162.0 hours), and ECMO flows (including BSA-indexed flows) remained similar between 4 and 24 hours after cannulation. Before BAS, 27 patients (96.4%) were receiving at least 1 inotropic agent. BAS was performed a median of 14 hours (Q1-Q3: 4.5-22.0 hours) after ECMO initiation using a median balloon diameter of 14 mm (Q1-Q3: 14-15 mm). Septostomy was performed under transesophageal echocardiographic guidance in 82.1% of patients and intracardiac echocardiographic guidance in 17.9%. Intraprocedural shunt creation was frequently confirmed by Doppler echocardiography (89.3%), whereas invasive hemodynamic confirmation was documented in 46.4% of procedures. The method of intraprocedural confirmation was not documented in 2 patients (7.1%).

**Table 2:** VA-ECMO Support and Balloon Atrial Septostomy Procedural Characteristics by LV Venting Failure Status

	Overall Cohort (N = 28)	LV Venting Failure (n = 15)	No LV Venting Failure (n = 13)
ECMO characteristics			
ECMO run time, h	79.0 (54.5-162.0)	119.0 (72.2-192.2)	69.0 (45.5-86.5)
Flow at 4 h, L/min	3.1 (2.5-3.7)	3.1 (2.8-4.0)	3.2 (2.4-3.6)
Flow at 24 h, L/min	3.3 (2.8-3.6)	3.2 (2.8-3.6)	3.3 (2.7-3.6)
Indexed flow at 4 h, L/min/m^2^	1.55 (1.35-1.84)	1.58 (1.51-1.88)	1.52 (1.30-1.80)
Indexed flow at 24 h, L/min/m^2^	1.63 (1.39-1.77)	1.58 (1.39-1.77)	1.68 (1.39-1.77)
Antiplatelet therapy during run	6 (21.4%)	3 (20.0%)	3 (23.1%)
Differential hypoxemia	0 (0.0%)	0 (0.0%)	0 (0.0%)
BAS procedural characteristics			
Time to BAS, h	14 (4.5-22)	14 (2-22)	14 (5-47.5)
Inotropic support before BAS	27 (96.4%)	14 (93.3%)	13 (100.0%)
Balloon diameter, mm	14 (14-15)	14 (14-16)	14 (13-15)
ICE	5 (17.9%)	2 (13.3%)	3 (23.1%)
TEE	23 (82.1%)	13 (86.7%)	10 (76.9%)
Technical success confirmation			
Doppler echocardiography	25 (89.3%)	13 (86.7%)	12 (92.3%)
Hemodynamics	13 (46.4%)	7 (46.7%)	6 (46.2%)
Not documented	2 (7.1%)	1 (6.7%)	1 (7.7%)

Values are median (Q1-Q3) or n (%). Values are presented overall and according to eventual LV venting-failure status. Circuit flow was measured at 4 and 24 hours after VA-ECMO cannulation and indexed to body surface area. Time to BAS was calculated from VA-ECMO initiation. Methods used to confirm procedural success were not mutually exclusive.

BAS = balloon atrial septostomy; ICE = intracardiac echocardiography; TEE = transesophageal echocardiography; other abbreviations as in Table 1.

### Laboratory and hemodynamic trends

Laboratory trajectories for selected markers, stratified by LV venting failure status, are shown in Supplemental Figure 2. Hemodynamic trends are displayed in Figure 1. There were no significant differences in trajectories of mean arterial pressure (MAP), pulmonary artery (PA) systolic pressure, PA diastolic pressure, or heart rate between patients who experienced LV venting failure and those who did not. Across the overall cohort, MAP increased over time following ECMO initiation, but the magnitude and pattern of change were similar between groups. Although patients with LV venting failure demonstrated higher baseline heart rates, subsequent heart rate trends after ECMO cannulation did not differ significantly from those without venting failure.

**Figure 1:**
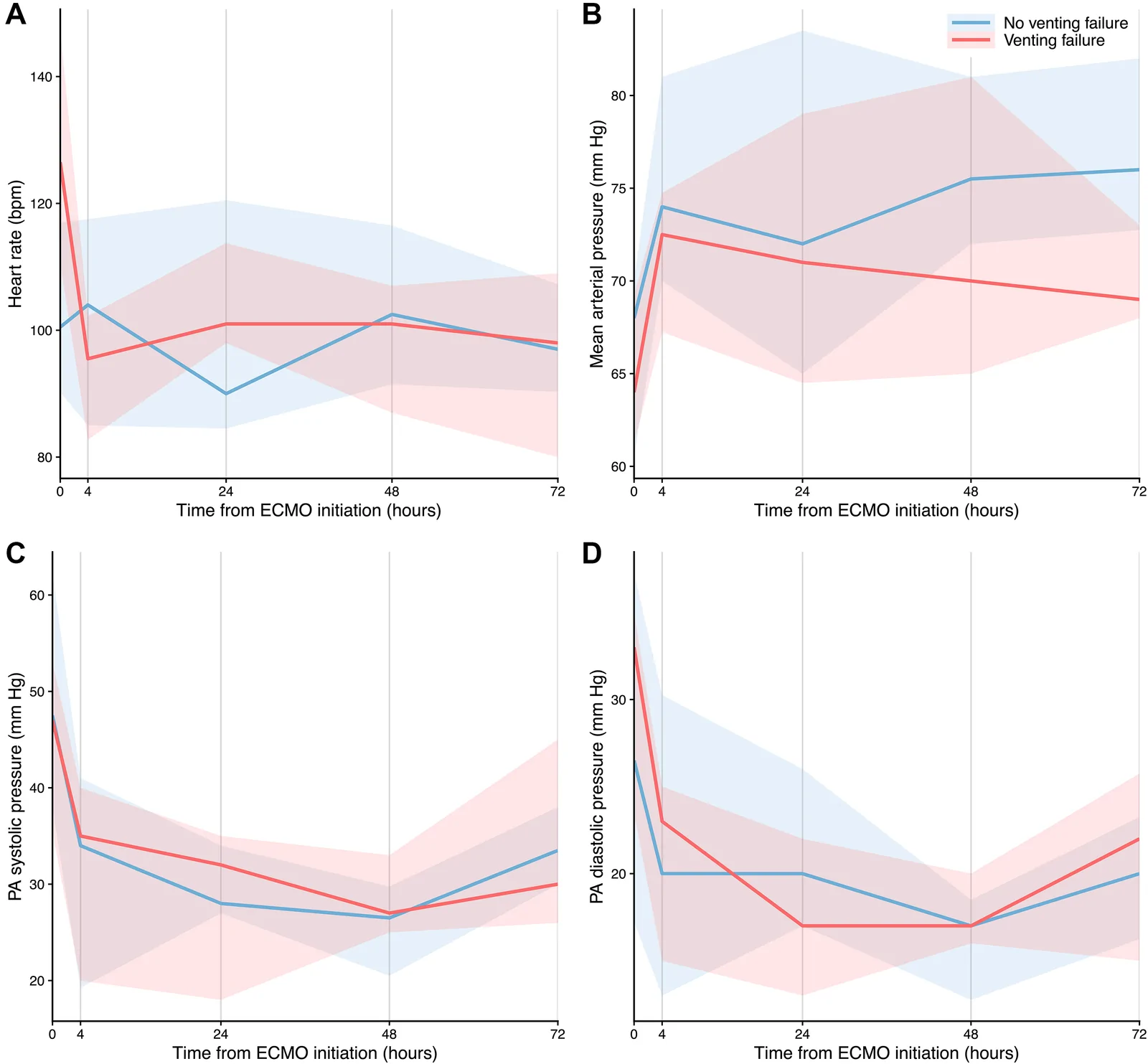
Hemodynamic Trajectories by Eventual LV Venting Failure Status Serial hemodynamic measurements are shown at baseline and 4, 24, 48, and 72 hours after VA-ECMO initiation for heart rate (A), mean arterial pressure (B), pulmonary artery systolic pressure (C), and pulmonary artery diastolic pressure (D) stratified according to eventual LV venting-failure status. Lines indicate medians, and shaded regions indicate IQRs. bpm = beats per minute; ECMO = extracorporeal membrane oxygenation; LV = left ventricular; PA = pulmonary artery; VA = venoarterial.

Among 21 patients with paired hemodynamic measurements, BAS was associated with a significant reduction in mLAP (Figure 2). The median mLAP decreased from 23 mm Hg (Q1-Q3: 12-28 mm Hg) before septostomy to 13 mm Hg (Q1-Q3: 10-20 mm Hg) after the procedure (*P* < 0.001). The cohort mean mLAP decreased from 20.5 mm Hg (SD: 10.9) to 13.5 mm Hg (SD: 7.2), and the paired Student's *t*-test sensitivity analysis produced a concordant result (*P* < 0.001).

**Figure 2:**
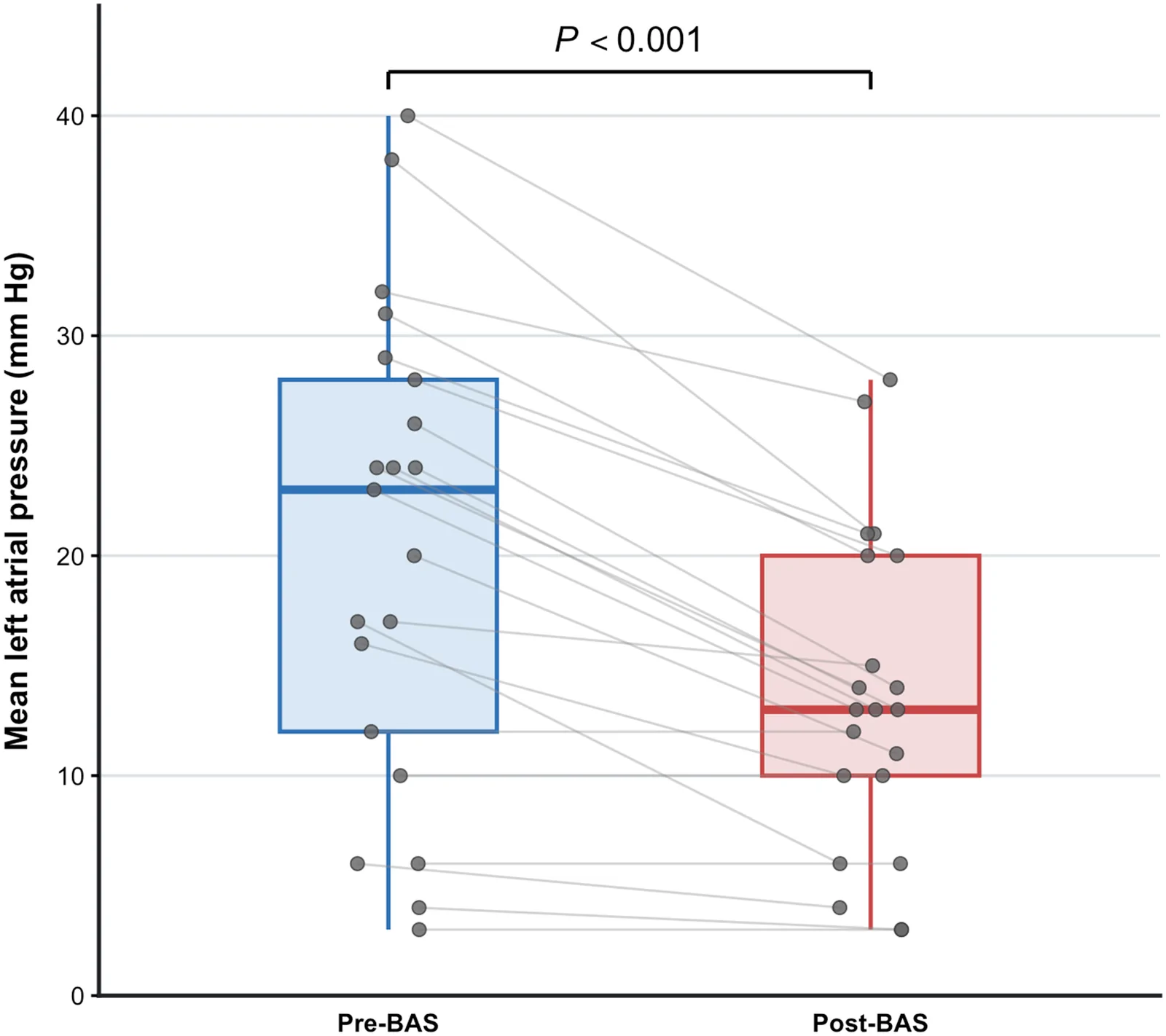
Left Atrial Pressure Reduction Following BAS Paired patient-level mean left atrial pressure measurements from 21 patients before and after balloon atrial septostomy. Points represent individual measurements, and gray lines connect measurements from the same patient. Horizontal lines within the boxes indicate medians, boxes indicate IQRs, and whiskers extend to the most extreme observations within 1.5 times the IQR. The *P* value was calculated using the 2-sided Wilcoxon signed rank test. BAS = balloon atrial septostomy.

### Pulse pressure

In the exploratory outcome-stratified analysis, observed arterial pulse pressure trajectories differed according to eventual LV venting failure status (venting failure × time interaction: F = 3.15, *P* = 0.018) (Figure 3). Pulse pressure declined early following VA-ECMO initiation in both groups. However, patients without LV venting failure had higher pulse pressure at 48 and 72 hours, whereas those with venting failure had lower pulse pressure at later time points. Model-estimated between-group differences (venting failure minus no venting failure) were −20.8 mm Hg at 48 hours (Holm-adjusted *P* = 0.010) and −18.1 mm Hg at 72 hours (Holm-adjusted *P* = 0.031), with no significant differences observed at baseline or within the first 24 hours following ECMO initiation. Sensitivity analysis excluding 4 patients who died within 30 days without documented LV venting failure yielded similar results (failure × time interaction *P* = 0.026), with higher model-estimated pulse pressure among patients without failure at 48 hours (difference: 24.8 mm Hg; Holm-adjusted *P* = 0.003) and 72 hours (difference: 22.7 mm Hg; Holm-adjusted *P* = 0.007).

**Figure 3:**
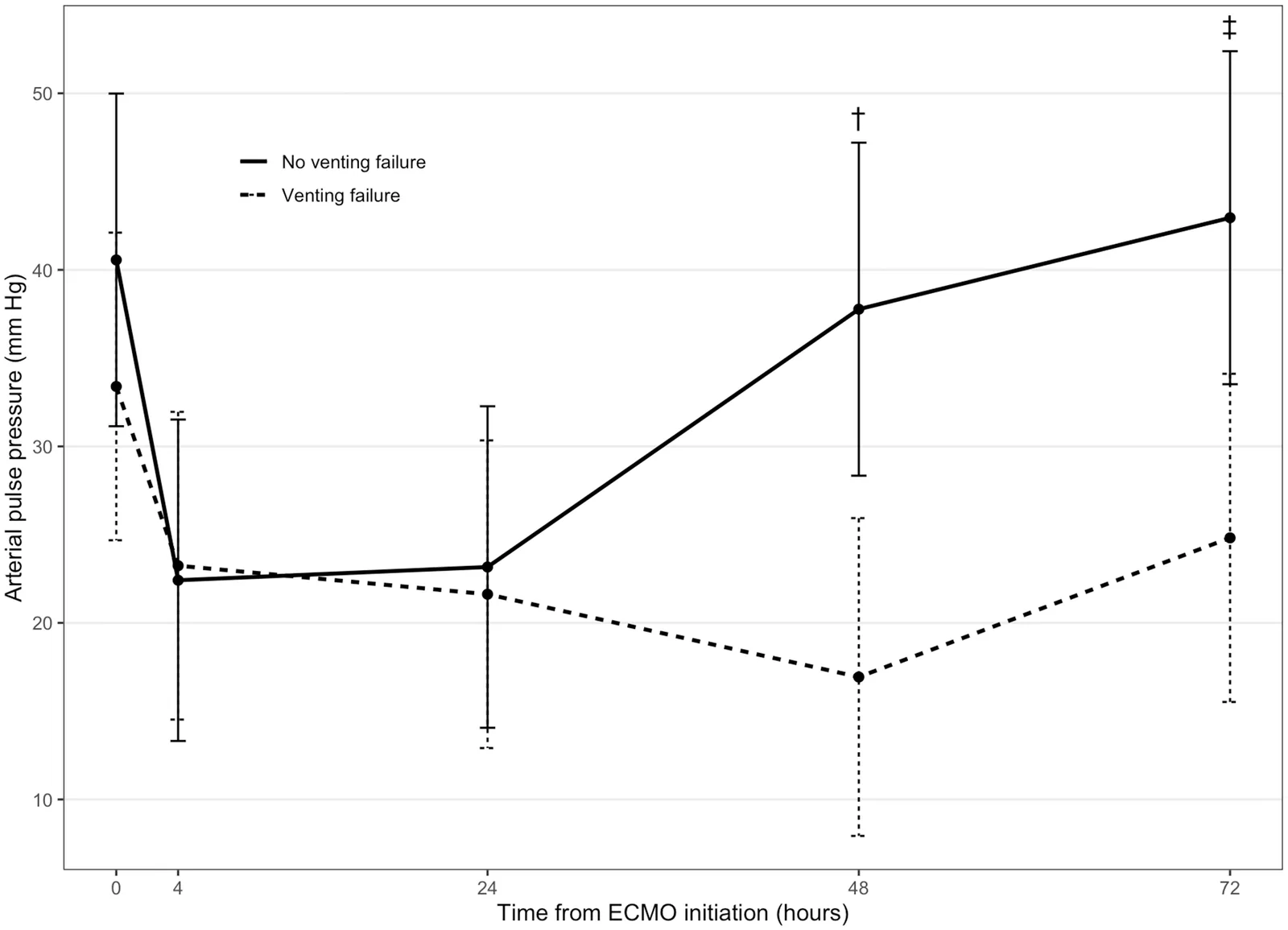
Arterial Pulse Pressure Trajectories by Eventual LV Venting Failure Status Model-estimated marginal means and 95% CIs for arterial pulse pressure are shown at baseline and 4, 24, 48, and 72 hours after VA-ECMO initiation. Baseline was defined as the lowest arterial pulse pressure recorded during the 24 hours preceding VA-ECMO initiation. The solid line represents patients who ultimately did not experience the composite LV venting-failure endpoint, and the dashed line represents those who did. Error bars indicate 95% CIs. Between-group contrasts were significant at 48 hours (†Holm-adjusted *P* = 0.010) and 72 hours (‡Holm-adjusted *P* = 0.031). These estimates describe differences between outcome-defined groups. Abbreviations as in Figure 1.

### Outcomes

Survival to hospital discharge occurred in 15 patients (53.6%), while 30-day mortality was 42.9% (Central Illustration). Despite septostomy, the composite outcome of LV venting failure occurred in 53.6% of the cohort. The most frequent components of venting failure were pulmonary edema (39.3%), echocardiographic evidence of LV distension (35.7%), and SEC in the LV or aortic root (32.1%). Thrombotic complications were less common, including LV thrombus in 14.3% and aortic root thrombus in 3.6%, with no cerebrovascular events observed. Escalation to additional mechanical unloading with Impella occurred in 10.7% of patients, whereas no patients required escalation to intra-aortic balloon pump. The median time to pulmonary edema after septostomy was 21.0 hours (Q1-Q3: 6.4-35.6 hours). Of the patients who underwent escalation of mechanical support, 2 were successfully weaned from VA-ECMO and decannulated, whereas 1 underwent heart transplantation. Among the 13 patients who did not meet the composite LV venting-failure endpoint, 4 (30.8%) died within 30 days. These deaths occurred a median of 90.9 hours (Q1-Q3: 68.2-116.2 hours) after VA-ECMO initiation.

**Central Illustration:**
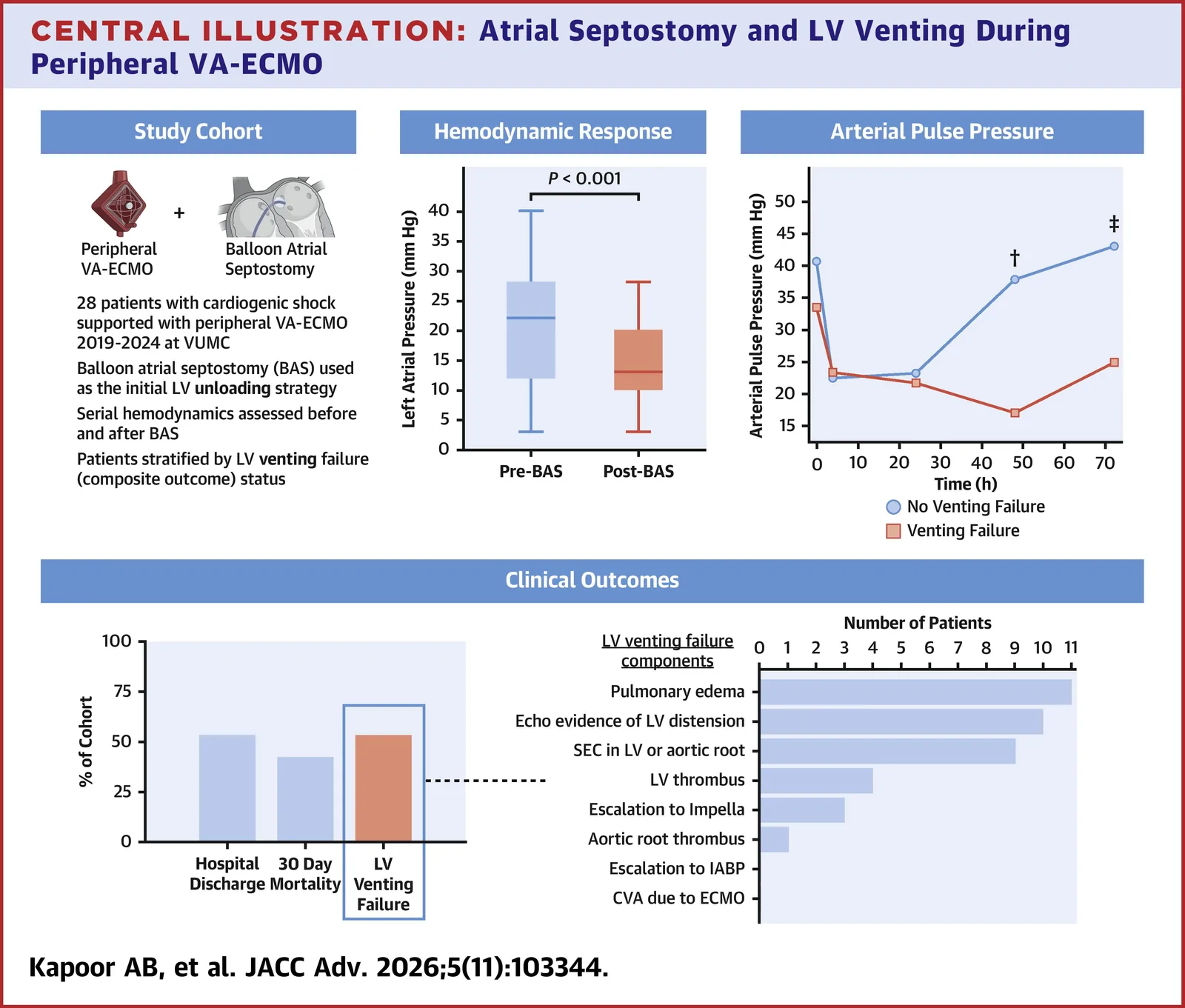
Atrial Septostomy and LV Venting During Peripheral VA-ECMO Balloon atrial septostomy was associated with a significant reduction in left atrial pressure, although LV venting failure remained common. Pulse pressure was significantly higher at 48 hours (†*P* = 0.010) and 72 hours (‡*P* = 0.031) among patients who ultimately did not experience LV venting failure compared with those who did. The lower panels summarize clinical outcomes and the individual components of the composite venting-failure endpoint. Components of the composite outcome were not mutually exclusive. CVA = cerebrovascular accident; ECMO = extracorporeal membrane oxygenation; IABP = intra-aortic balloon pump; LV = left ventricular; SEC = spontaneous echocardiographic contrast; VA = venoarterial; VUMC = Vanderbilt University Medical Center.

## Discussion

In this single-center retrospective analysis of adult CS patients supported with peripheral VA-ECMO, we found that although BAS significantly reduced mLAP, LV venting failure occurred in 53.6% of patients. Consistent with prior observational studies demonstrating reductions in left-sided filling pressures following BAS, our data suggest that BAS is an effective atrial-level unloading strategy during VA-ECMO.^5^, ^6^, ^7^, ^8^ However, this unloading does not translate to durable LV venting in a substantial proportion of patients. As such, our study extends the current published reports by using granular hemodynamic data and clinically relevant longitudinal outcomes to assess the degree to which successful LV unloading through BAS translates into durable LV venting.^4^^,^^9^^,^^10^ Specifically, we found that most patients met the composite endpoint for LV venting failure due to recurrent pulmonary edema or echocardiographic markers of LV distension/stasis, indicating that BAS does not always prevent LV stasis during VA-ECMO.^1^^,^^2^^,^^11^

The variability in durable LV venting observed in this cohort is mechanistically consistent with the physiologic limitations of atrial-level shunting during VA-ECMO. BAS lowers LA and LV end-diastolic pressures by facilitating interatrial shunting, decompressing the pulmonary venous circulation and reducing hydrostatic pulmonary congestion.^6^, ^7^, ^8^^,^^12^ In patients with sufficient residual LV contractility or lower ECMO-mediated afterload, this pressure relief may be adequate to promote LV emptying and mitigate stasis. In contrast, patients with little to no native myocardial contractility are likely to experience intracavitary stasis despite LA decompression.^11^^,^^13^ As a result, the pressure-lowering benefits of atrial shunting may be insufficient to overcome ECMO-mediated afterload, particularly when native LV contractility is severely depressed.^8^^,^^14^ In addition, procedural and anatomic heterogeneity may have impacted shunt adequacy. The degree of septal dilation, balloon size selection, and confirmation of procedural success were not standardized across the study period due to its retrospective nature and provider variability. These findings suggest that BAS occupies an intermediate position along the unloading spectrum: capable of achieving effective venting in a meaningful subset of patients, but insufficient as a standalone strategy in others.^2^^,^^15^ Percutaneous PA venting represents another potential indirect unloading strategy that reduces LV preload.^16^^,^^17^ However, because PA drainage does not generate antegrade transaortic flow, it may not ensure adequate LV or aortic root washout when native LV ejection and aortic valve opening are absent, potentially leaving residual risk of stasis.

Arterial pulse pressure provided a dynamic physiologic measure of native LV ejection during VA-ECMO support. Although pulse pressure is derived from arterial pressure waveforms, it reflects native LV stroke volume and aortic valve opening rather than systemic pressure targets alone.^1^^,^^18^ Pulse pressure declined early after ECMO initiation in both groups, consistent with the increase in LV afterload imposed by retrograde flow. After reaching their respective nadirs, model-estimated pulse pressure increased in both groups; however, patients who ultimately did not experience LV venting failure had significantly higher pulse pressure at both 48 and 72 hours. In contrast, patients who experienced venting failure demonstrated persistently attenuated pulse pressure trajectories. Because these groups were defined according to eventual LV venting-failure status, the observed differences should be interpreted as descriptive. Although pulse pressure has not been prospectively validated as a predictor of LV venting failure, the observed separation in longitudinal trajectories supports prospective evaluation of pulse pressure as a potential physiologic correlate of sustained native LV ejection following atrial-level unloading.

Laboratory biomarkers provided complementary insights into physiologic trajectories after VA-ECMO initiation and early BAS. Lactate and bilirubin declined over time across the cohort, consistent with early improvement in global perfusion during VA-ECMO support, and these trends were observed irrespective of subsequent LV venting adequacy. Given that most patients underwent BAS soon after ECMO initiation, these findings likely reflect early physiologic stabilization during combined ECMO support and atrial-level decompression rather than isolated effects of venting efficacy. Prior VA-ECMO studies have reported heterogeneous lactate and bilirubin trajectories and have emphasized these biomarkers primarily as prognostic indicators; the more uniform decline observed here may reflect differences in cohort selection, timing of assessment, and early intervention.^19^, ^20^, ^21^ Hepatic transaminases exhibited outcome-dependent trajectories, with aspartate aminotransferase and alanine transaminase evolving differently in patients with vs without LV venting failure. These divergent patterns likely reflect heterogeneity in baseline hepatic injury burden and underlying clinical trajectories rather than a direct effect of venting efficacy. Notably, macrohemodynamic parameters, including MAP, PA pressures, and heart rate, did not differ by venting outcome, underscoring a dissociation between conventional hemodynamic measures and organ-level injury markers. Together, these findings indicate that laboratory biomarkers and macrohemodynamic parameters may not reliably reflect venting adequacy, which was more clearly distinguished by clinically meaningful outcomes.

The clinical outcomes in this cohort provide important context for the physiologic effects of BAS during peripheral VA-ECMO support. Although effective LV venting was achieved in a substantial subset of patients, clinically significant venting failure remained common. Venting failure was driven predominantly by pulmonary edema and echocardiographic LV distension or SEC, rather than thromboembolic events or early escalation to an additional venting strategy. These patterns suggest that atrial-level decompression may be sufficient to prevent adverse sequelae of LV stasis in select patients but does not uniformly protect against congestion-related complications during ongoing ECMO support. It is reasonable to hypothesize that atrial-level decompression as an LV venting strategy may be most effective in a subset of patients with preserved intrinsic myocardial contractility, with maintained pulse pressure representing a potential physiologic correlate. Larger prospective studies incorporating clinical, hemodynamic, and echocardiographic parameters are needed to identify patients in whom BAS alone provides adequate LV venting. Survival to hospital discharge and early mortality rates further underscore the high-risk nature of patients selected for BAS and emphasize that successful venting represents a necessary but not sufficient determinant of short-term clinical outcomes. Therefore, although BAS represents a feasible and effective venting approach in select patients, its use should be accompanied by close physiologic and imaging-based monitoring, and its role should be evaluated in head-to-head comparisons with alternative unloading strategies that more directly mitigate LV afterload during VA-ECMO support.

### Study limitations

First, its single-center, retrospective design and sample size limit statistical power and generalizability. Second, selection bias is possible, as patients undergoing BAS likely represent a subgroup with more severe hemodynamic compromise or higher anticipated risk of LV distension, which may overestimate rates of venting failure. Third, although all patients underwent BAS, heterogeneity in procedural technique—including variation in balloon size—may have influenced procedural efficacy. Fourth, although ECMO circuit flows were retrospectively indexed to BSA at 4 and 24 hours, they were clinically titrated rather than targeted to a prespecified flow index. Total cardiac index could not be reliably quantified during VA-ECMO support. In addition, the effects of cannula configuration and circuit drainage pressures on LV unloading were not systematically evaluated. Fifth, this study was not designed to compare BAS with direct mechanical LV unloading using a microaxial transvalvular pump (Impella). Escalation to Impella was incorporated into the definition of BAS failure and therefore could not be evaluated as an independent comparator. In addition, early death may have shortened the opportunity to observe LV venting failure in some patients. Furthermore, because LV venting-failure status was ascertained during follow-up, the outcome-stratified longitudinal analyses cannot establish temporality and may be affected by reverse causation. In particular, later physiologic measurements may characterize the clinical course after failure rather than predict its occurrence. These analyses should therefore be interpreted as exploratory and descriptive. Comparative studies are needed to determine the relative effectiveness, safety, and appropriate patient selection for BAS-first vs device-based unloading strategies. Finally, although we incorporated serial hemodynamic, laboratory, and echocardiographic data, outcomes were largely physiologic and short-term; the study was not designed to assess longer-term endpoints such as myocardial recovery.

## Conclusions

In patients with CS supported with peripheral VA-ECMO, BAS provides LA decompression, as evidenced by significant reductions in measured mLAP. However, the durability of LV venting achieved with atrial-level decompression alone is variable, with LV venting failure occurring in approximately half of patients. These findings underscore that although BAS represents a feasible LV venting strategy in select individuals, it requires close physiologic and imaging-based surveillance to detect ongoing venting failure. Observed longitudinal arterial pulse pressure trajectories may provide complementary physiologic insight into native LV ejection following atrial-level unloading during VA-ECMO support. Future studies comparing atrial septostomy with alternative unloading strategies and incorporating enhanced surveillance are needed to define optimal patient selection and management during VA-ECMO support.

Perspectives **COMPETENCY IN MEDICAL KNOWLEDGE:** BAS can achieve LA decompression during peripheral VA-ECMO, but atrial-level unloading does not uniformly provide durable LV venting. Continued physiologic and echocardiographic surveillance is therefore important to identify persistent pulmonary congestion, LV distension, or cardiac stasis following septostomy.

**TRANSLATIONAL OUTLOOK:** Prospective studies should compare atrial septostomy with alternative LV unloading strategies, standardize assessment of procedural efficacy, and determine whether serial pulse pressure can identify patients who require escalation of LV venting.

## Funding support and author disclosures

Dr Rali is a consultant for Abiomed and Abbott. All other authors have reported that they have no relationships relevant to the contents of this paper to disclose.
