# Supplementary material for: Atrial Septostomy and Left Ventricular Venting During Peripheral VA-ECMO
Source: JACC Adv. 2026 Oct 17;5(11):103344. doi: 10.1016/j.jacadv.2026.103344 (PMC13696345; doi:10.1016/j.jacadv.2026.103344)
Supplement: Supplementary file 1 [file mmc1.pdf]

**Study Cohort Derivation: VA-ECMO Patients Undergoing LV Venting with BAS**

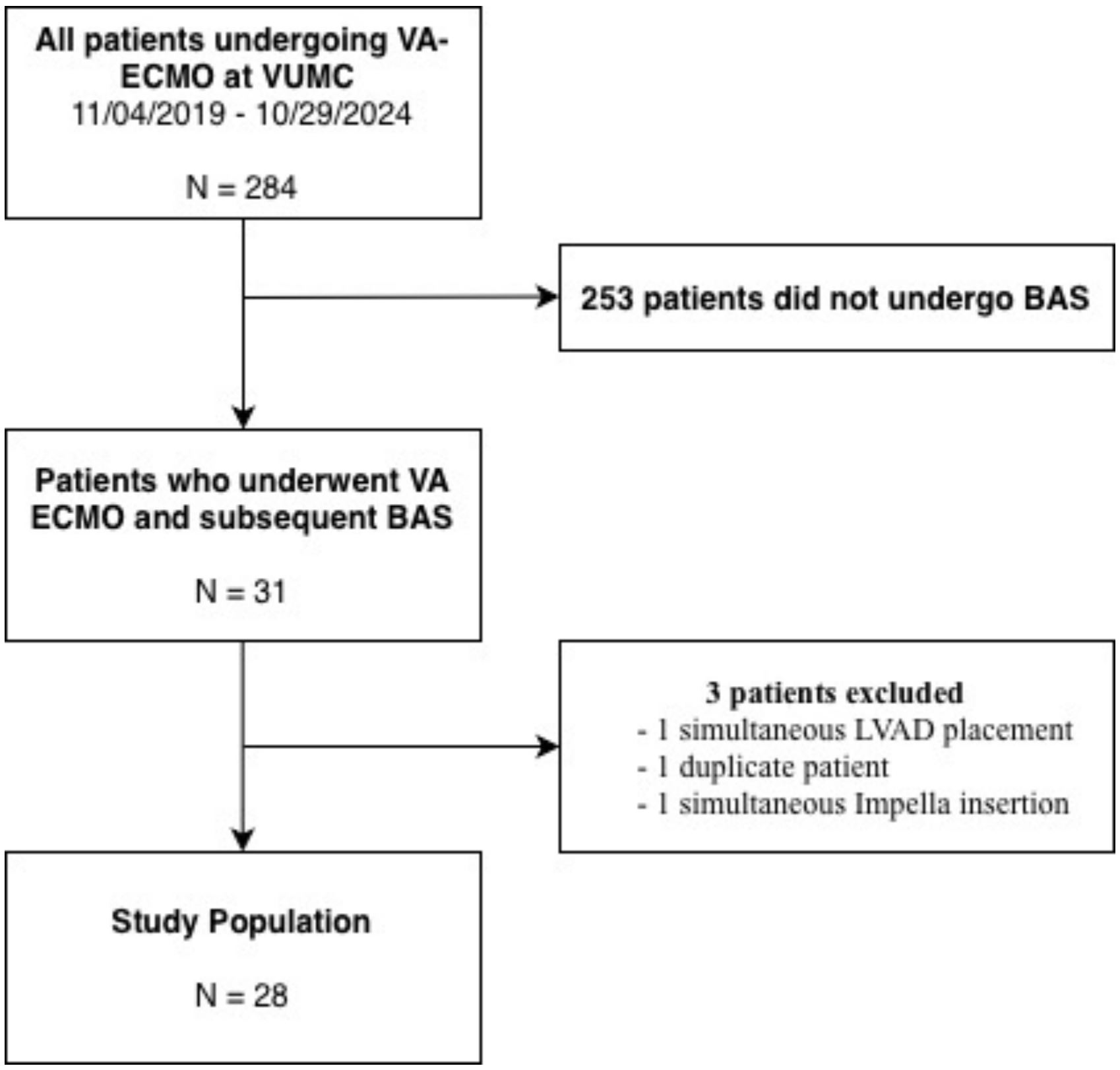


**Supplemental Figure 1**: Patient selection for adults supported with VA-ECMO who underwent balloon atrial septostomy (BAS) for LV venting at VUMC (2019–2024). Of 31 eligible patients, 3 were excluded (simultaneous LVAD/Impella, duplicate), resulting in a final cohort of 28.

**Temporal Trends in Laboratory Markers Following VA-ECMO Initiation by LV Venting Failure**

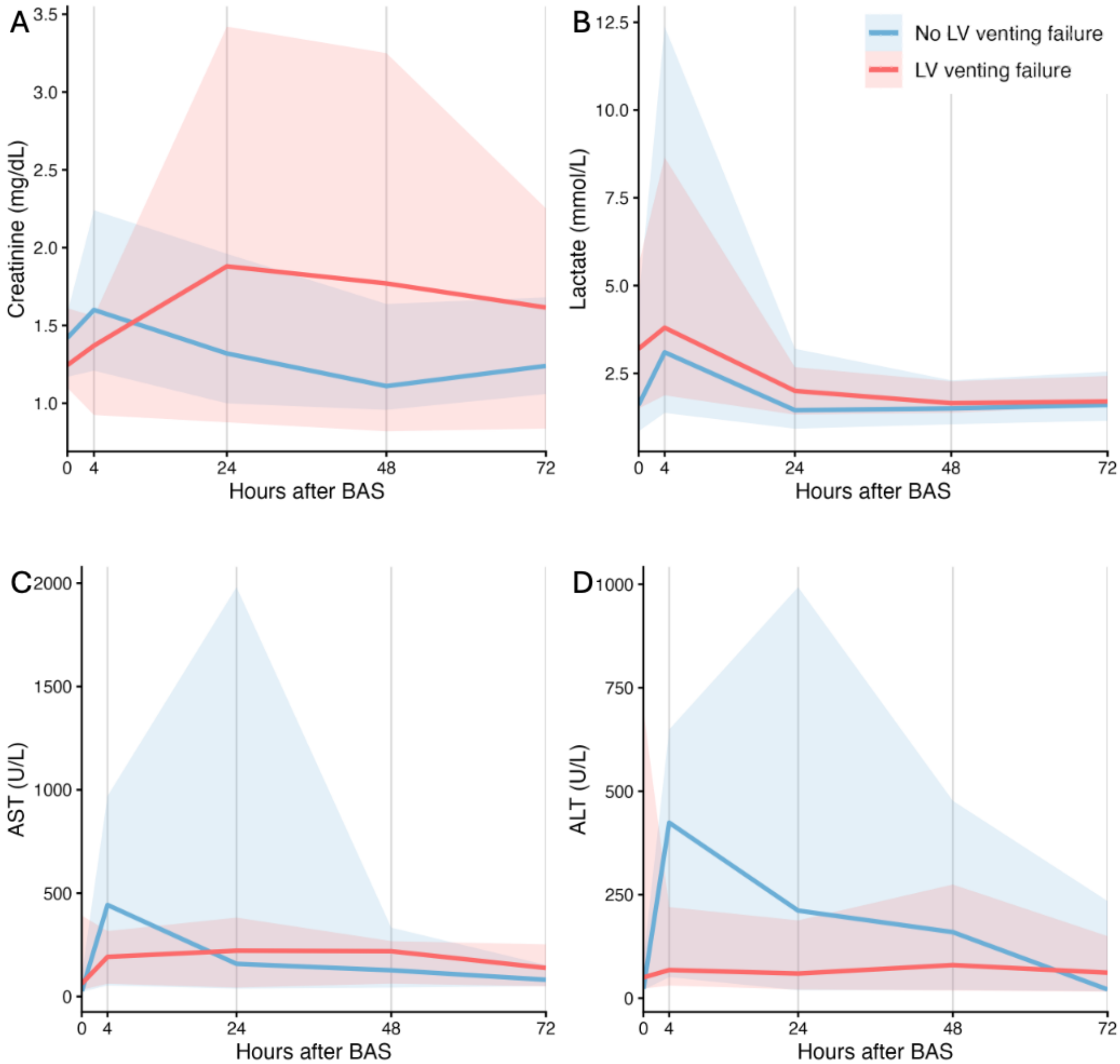


**Supplemental Figure 2**: Serial laboratory trajectories are shown for (A) creatinine, (B) lactate, (C) aspartate aminotransferase (AST), and (D) alanine aminotransferase (ALT). Time points are referenced to VA-ECMO initiation (0, 4, 24, 48, and 72 hours). Solid lines represent group medians, and shaded bands denote interquartile ranges. Patients are stratified by LV venting outcome, defined by the presence (red) or absence (blue) of the composite LV venting failure endpoint.
